# The Diagnosis of Hydrophobia by Rapid Examination

**Published:** 1902-03-22

**Authors:** 


### THE DIAGNOSIS OF HYDROPHOBIA BY RAPID EXAMINATION.

A number of valuable contributions to the pathological appearances in rabies have been published by von Babes of Bucharest and by Professor van Gehuchten and others. In a paper published in *La Press Medicale* (March 7th 1900), van Gehuchten and Nelis refer to the work of previous observers regarding the minute histological changes occurring in the medulla oblongata of dogs and other animals afflicted with rabies, but maintain that such changes are not of diagnostic value. Van Gehuchten and Nelis, however, claim to have found changes in the ganglia of the cranial, spinal, and sympathetic nerves which they regard as peculiar to rabies and of special diagnostic value therefore in doubtful cases. Thus, apart from the general signs of severe toxæmia of the nervous system, viz., the intense general vascular congestion and hyperæmia of the brain and spinal cord observed in fatal cases with the naked eye, and the microscopic appearances of a perivascular infiltration and increase of small nucleated cells throughout the substance of the brain and spinal cord, other and more characteristic changes occur as follows:—The connective-tissue cells which surround the nerve cells in the sympathetic and spinal ganglia (and called endothelial capsule-cells) increase and multiply in large numbers and produce a partial or total destruction of the enclosed ganglionic nerve cell. In some place the destruction and disappearance of the nerve cell is complete, and its place is occupied by a mass or nodule of closely packed small round cells in mulberry-shaped aggregations. The partially damaged ganglion cells themselves show various shapes of chromatolysis and of disintegration, though these intra-cellular changes are not of absolute diagnostic value. In several rabbits and dogs which had died of hydrophobia, these changes in the endothelial cells were always met with in striking degree, and in animals inoculated with rabies such changes could be easily reproduced, thus leaving no doubt in the mind as to their connection with the hydrophobic poison. In two cases of rabies affecting the human being and ending fatally, similar changes were found. In another paper, read before the Paris Académie de Médecine, contributed by Babes, of Bucharest, it was stated that the old method of inoculation of the medulla gave a positive diagnosis, though a delay of a fortnight or three weeks was needed before the symptoms appeared fully and the diagnosis was made positive. However, to avoid such undue delay, Babes has practised the following method: All dogs affected with rabies who have bitten men or animals are killed and portions of the brain prepared and injected into rabbits by the intracranial method. Further, a portion of the medulla oblongata is cut and stained with methyl blue and carbol fuchsin. The suspected dog may be regarded as having suffered with rabies if microscopic examination of the sections shows nodular masses of small connective-tissue cells surrounding or replacing nerve-cells, and if groups of leucocytes are distributed along the course of the perivascular channels. In 487 suspected dogs portions of the bulb were removed and inoculations made into rabbits, with the result that 410 of the rabbits died of hydrophobia in from one to five weeks after the inoculation. In 384 cases microscopic examination of the medulla gave evidences of rabies, and all the rabbits inoculated from these also gave signs of hydrophobia. Babes does not, however, accept the findings of van Gebuchten as of real diagnostic value, but at another meeting of the Académie de Médecine, M. Nocard mentioned that experiments were being carried out at the Veterinary School of Toulouse by MM. Caillé and

Vallée, and that in examining nine dogs which had died of hydrophobia the changes described by van Gebuchten and Nelis were found in the endothelial capsule-cells and in the ganglionic nerve cells. In order, however, to ascertain whether these pericellular lesions and changes were produced early or late they inoculated six dogs, each with half a cubic centimetre of the emulsion from the brain of a fatal canine case of rabies. Three of the six developed rabies and were killed soon after the appearance of the earliest symptoms, but in only one of these were the lesions present to any characteristic extent, thus showing that the lesion was rather late in appearing in its characteristic form.

